# The Sources and Potential Hosts Identification of Antibiotic Resistance Genes in the Yellow River, Revealed by Metagenomic Analysis

**DOI:** 10.3390/ijerph191610420

**Published:** 2022-08-21

**Authors:** Kai Zhang, Kuangjia Li, Ziyi Liu, Qidi Li, Wenpeng Li, Qi Chen, Yangchun Xia, Feiyue Hu, Fengxia Yang

**Affiliations:** 1 Henan Key Laboratory for Synergistic Prevention of Water and Soil Environmental Pollution, School of Geographic Sciences, Xinyang Normal University, Xinyang 464000, China; 2 Development Research Center, Ministry of Water Resources of People’s Republic of China, Beijing 100032, China; 3 School of Marine Science and Technology, Tianjin University, Tianjin 300072, China; 4 Agro-Environmental Protection Institute, Ministry of Agriculture and Rural Affairs, Tianjin 300191, China

**Keywords:** antibiotic resistance genes, host identification, microbial sources tracking, Yellow River

## Abstract

The fate of antibiotic resistance genes (ARGs) has been revealed in various environmental media in recent years. Namely, the emergence of genes that resist colistin and carbapenems has attracted wide attention. However, the pollution condition of ARGs and sources in the Yellow River is still little understood, despite the river being the second longest in China. The present study determined the levels of ARG pollution in the Henan section of the Yellow River and evaluated the role of the aquaculture industry in the spread of ARGs. As revealed by the results, a total of 9 types of ARGs were detected in the sediments of the Yellow River, and the total ARG content in the Yellow River ranges from 7.27 to 245.45 RPKM. *Sul*1 and *sul*2 are the dominant ARGs, and the huge usage of sulfonamides, horizontal gene transfer, and wide bacteria host contribute to the prevalence of these two genes. The results of Spearman correlation analysis indicate that the breeding industry has little influence on ARGs in the Yellow River. Network analysis reveals that the opportunistic pathogen *Pseudomonas* is the potential host of *sul*1, *tet*G, and *ANT*(3′′)-IIa, which can pose a risk to human health.

## 1. Introduction

Since the 21st century, antibiotic resistance genes (ARGs) have been continuously reported [1]. ARGs in the environment could transfer to pathogens and help them against antibiotics, making it more difficult to control bacterial resistance. Currently, ARGs have been regarded as an emerging pollutant [2,3,4]. The distribution characteristic and the level of ARGs pollution in the natural environment, such as rivers, lakes, reservoirs, marine, and even drinking water, have been widely studied [5,6,7,8]. The results showed that ARG pollution existed to different degrees in the environment. Especially the emergence of genes that resist colistin antibiotics, which is regarded as the last resort antibiotic, has attracted widespread attention [9,10,11].

Antibiotics could induce ARGs by a selection or co-selection mechanism [12]. Heavy metals could drive the propagation of ARGs through a co-selection mechanism [13,14]. Moreover, ionic liquid, polycyclic aromatic hydrocarbons (PAHs), pesticides, chlorination, and even microplastics have been proven to contribute to the dissemination of ARGs in the environment [15,16,17]. In particular, fecal pollution sources are considered to be one of the important sources of ARG in the environment [18,19]. The fecal indicator bacteria (FIB) and microbial sources tracking (MST) method are used to indicate fecal pollution levels. However, FIB generally had poor relationships with health outcomes and could not provide information regarding the source(s) of contamination, and this limitation of FIB led to the emergence of MST [20]. The microbial sources tracking (MST) method can be used for determining the pollution sources by analyzing the relationship between certain fecal microorganisms and genes with a specific host [21]. Recently, the MST method has been used to identify the fecal pollution sources of ARGs in the water system [22,23]. However, as an emerging pollutant, the fecal source tracking of ARGs by MST is still in its infancy, and the role of MST should be further determined in the dissemination of ARGs.

The Yellow River is the second largest river in China and is also the most important river in North China, playing an important role in the daily lives of more than 155 million people [24]. The pollution levels of various pollutants, including antibiotics, heavy metals, PAHs, and endocrine disrupting chemicals, have been revealed in the Yellow River [25,26,27,28]. Recently, Yu et al. investigated the level of ARGs pollution in the Lanzhou section of the Yellow River by adopting high-throughput quantitative PCR techniques [29]. However, the ARGs pollution condition in the Yellow River is still little understood due to the long length of the river. Additionally, the main biological drivers of ARGs in the river are also unclear.

Henan province, which has been the number one province in total grain output since 2000, is the main agricultural province in China [30]. Furthermore, the breeding industry, as one of the main sources of ARGs, is developed in Henan province. However, China has introduced a plan named the National Action Plan to Combat Antimicrobial Resistance (2016–2020) to regulate ARGs pollution. The Chinese Ministry of Agriculture and Rural Affairs also introduced a series of policies, such as “National Action Plan to Combat Antimicrobial Resistance from Animal Resources (2017–2020)” and “MonitoringPlan for Antimicrobial Resistance from Animal Resources in 2019”, which requires reducing the antibiotics used in animal husbandry. Thus, the contribution of the breeding industry to the propagation of ARGs should be evaluated.

In summary, the ARG pollution pattern should be determined in the Henan section of the Yellow River. Furthermore, the role of the aquaculture industry in ARG dissemination needs to be clarified. This study quantified the antibiotic resistome, bacteria community and MST genes in the Yellow River by using metagenomic analysis, 16S rRNA amplicon sequencing, and real-time quantitative PCR (RT-qPCR), respectively. The objectives of this study were to (1) reveal the distribution pattern of the antibiotic resistome, (2) identify the main biotic drivers in the river, and (3) evaluate the role of fecal pollution sources in the propagation of ARGs in Yellow River sediments of the Henan section.

## 2. Materials and Methods

### 2.1. Sample Collection and DNA Extraction

**Figure 1:**
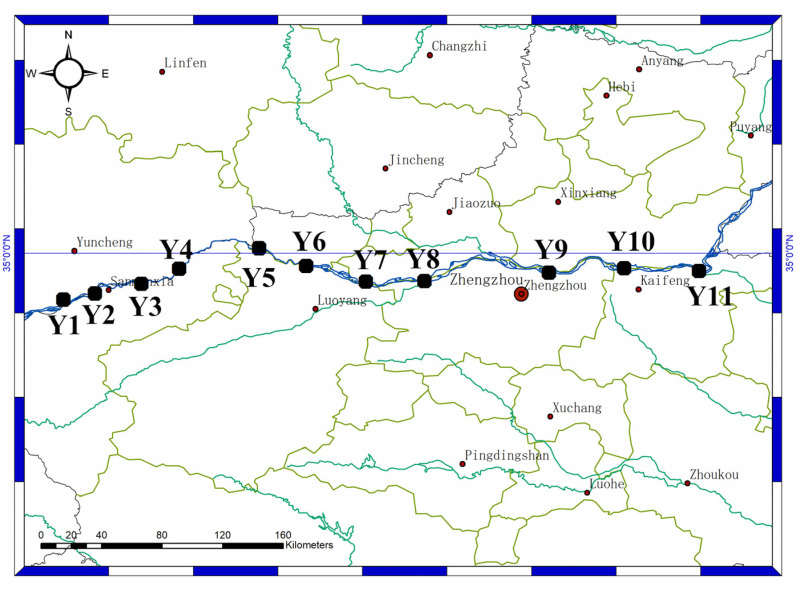
Sample site of the Yellow River in the Henan section.

The sampling was conducted from 28–29 January 2021. Eleven sediment samples were collected from the mainstream of the Yellow River in the Henan section (Figure 1). Sediment samples were collected with a sediment sampler (KH0201, Xinbao, Jintan, China) and stored in a Ziploc bag. The samples were sorted in an ice bath, transported to the laboratory immediately, and stored at −20 °C.

DNA extraction was conducted by using a DNA Extraction Kit (MP Biomedicals, Santa Ana, CA, USA) following the instructions manual. DNA quality was verified with 1.5% agarose gel electrophoresis. DNA yield and purity were verified by using a Nanodrop 2000 ultramicro spectrophotometer (Thermo Fisher, Waltham, MA, USA). The DNA content was verified by using a Qubit 4.0 Quantus Fluorometer (Thermo Fisher, Waltham, MA, USA).

### 2.2. Quantification of MST Indicators

In this study, a total of 7 MST indicators were determined, including mtDNA of horse, chicken, cow, human, dog, pig, and sheep. Moreover, 16Sr DNA was determined to calculate the relative abundance of MST indicators. All premiers of target genes are shown in Table S1. Quantification was conducted with a real-time qPCR instrument (ABI QuantStudio^®^3, Thermo Fisher Scientific, Waltham, MA, USA). The plasmids containing the MST indicator fragments were constructed with the pUC57 vector (Sangon Biotech, Shanghai, China) and used for the standard curves.

The 20 μL reactions contained 8.2 μL ddH_2_O, 0.4 μL forward and reverse primer, 10 μL SYBR Premix Ex TaqTM (Tli RNaseH Plus, TaKaRa, Japan), and 1.0 μL sediment DNA. The following reaction program was run as follows: initial denaturation (2 min at 95 °C), followed by 40 cycles consisting of 10 s at 95 °C, annealing (30 s at annealing temperature described in Table S2), extension (45 s at 72 °C), and a final extension (6 min at 72 °C). Each reaction was run in triplicate for each sample, and sterile water was used as the blank control.

### 2.3. 16S Amplicon Sequencing

The sediment microbial community was determined by adopting the Miseq PE300 platform. The bacteria-specific V3–V4 region of the 16S rRNA was amplified using the 338F (5′-ACTCCTACGGGAGGCAGCAG-3′) and 806R (5′-GGACTACHVGGGTWTCTAAT-3′) primers. All PCR reactions were carried out in a 20 μL system, which includes 5 × TransStart FastPfu buffer (4 μL), forward and reverse primer (0.8 μL for each), 2.5 mM dNTPs (2 μL), 0.4 μL DNA polymerase (TransStart FastPfu, Beijing, China), sediment DNA (10 ng), and ddH2O. Each amplification reaction was performed in triplicate in an ABI GeneAmp^®^ (Thermo Fisher, Waltham, MA, USA) 9700 instrument under the following program: initial denaturation (3 min at 95 °C), followed by 27 cycles consisting 10 s at 95 °C, annealing (30 s at 55 °C), extension (30 s at 72 °C), and a final extension (10 min at 72 °C). PCR products were examined by 2% agarose gel electrophoresis and then purified by an Axygen DNA Gel Extraction Kit. NEXTflexTM DNA-Seq Kit (Bioo Scientific, Austin, TX, USA) was used for constructing the library, then sequencing was performed at Majorbio Bio-Pharm Technology Co., Ltd. (Shanghai, China) (Majorbio). Sickle (available online: https://github.com/najoshi/sickle (accessed on 3 June 2011) was adopted to filter low-quality bases (length < 50 bp, quality value < 20 or presence of N bases). The operational taxonomy units (OTUs) were defined when the similarity of a representative read ≥ 97%. RDP classifier (available online: http://rdp.cme.msu.edu/ (accessed on 16 May 2012), version 2.2) was adopted to assign OTUs.

### 2.4. Metagenomic Analysis

The Covaris M220 ultrasonicator (Gene Company Limited, Hong Kong, China) was used for fragmenting DNA to approximately 400 bp. A paired-end (PE) library was constructed by adopting NEXTflexTM Rapid DNA-Seq (Bioo Scientific, Austin, TX, USA). Sequencing was conducted by adopting Illumina NovaSeq (Illumina, San Diego, CA, USA) at Majorbio. Fastp (available online: https://github.com/OpenGene/fastp (accessed on 12 February 2018), version 0.20.0) was used to filter low-quality reads (length < 50 bp, quality value < 20 or presence of N bases). Then sequences were then assembled to ≥300 bp contigs.

Resistome was characterized against the Comprehensive Antibiotic Resistance Database (CARD, version 3.0.9 website: http://arpcard.mcmaster.ca, accessed on 21 September 2017)) by adopting Diamond (version 0.8.35) with an e-value of ≤ 1 × 10^−5^ through the Majorbio cloud platform [31]). If the optimal hit of a sequence in the reference database exceeded 90% identity with ≥ 25 amino acids of alignment length, the sequence would be identified as an ARG-like ORF.

### 2.5. Statistical Analysis

All experimental data were statistically analyzed using Microsoft Excel 2019. Spearman correlation analysis was conducted by using SPSS V22.0 (IBM, Endicott, NY, USA) and was statistically significant when *p* < 0.05. The bacteria community of Y11 was not involved in any molecular biology experiment due to the low DNA content of this sample site.

## 3. Results

### 3.1. ARG Profile in the Sediments of the Yellow River

**Figure 2:**
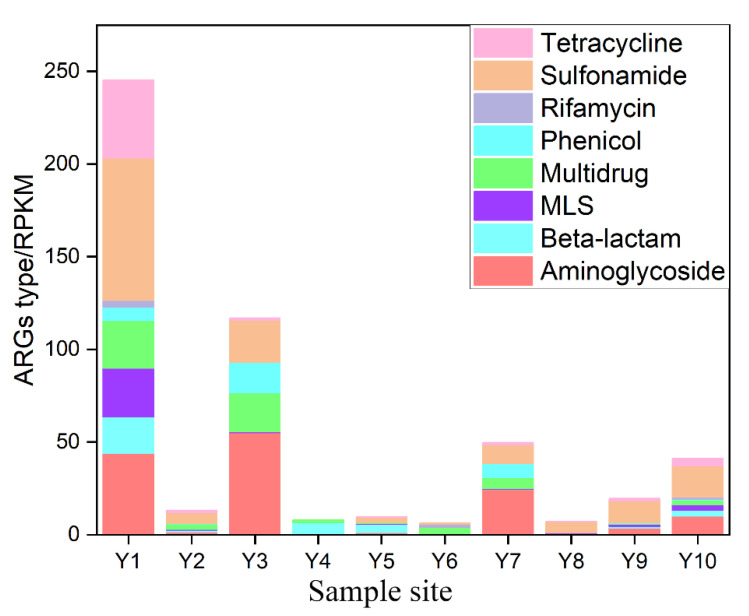
Abundance of detected ARG types in the sediment of the Yellow River.

A total of nine types of ARGs were detected in Yellow River sediments (Figure 2), among which sulfonamide resistance genes (*sul*-ARGs) were the dominant gene with an abundance of 15.39 PPM and a percentage of 33.73%, respectively. Sulfonamides (SAs) are one of the most widely used antibiotics, and a previous study reports that more than 20,000 tons of SAs are introduced into the biosphere each year [10]. Moreover, SAs are difficult to degrade, leading to the accumulation of this antibiotic in the environment [32,33]. The Aminoglycoside- and Multidrug-genes are the second and third highest gene types, with an average proportion of 18.35% and 15.90%, respectively. Aminoglycoside antibiotics have been used for nearly a century, and antibiotics have been widely used in China [34]. The antibiotic could easily dissolve in water and provide selective pressure for the corresponding ARGs. Moreover, the host of some Aminoglycoside genes has been proven wide, which is responsible for the high content of ARGs in the Yellow River [8,35].

The total ARG content in the Yellow River ranges from 7.27 to 245.45 RPKM, with a variation coefficient (CV) of 147.22%, indicating that there exists a large difference in the ARG content of the Yellow River. The highest value of ARGs is found in Y1 (245.45 RPKM), and the value is 2.09 times that of Y3, of which the ARGs content is the second highest. Y1 is the first sample site of Henan province and the closest point to Shanxi province. Previous studies investigating antibiotic levels in the Fen River, a typical tributary of the Yellow River in Shanxi, have shown higher levels of various antibiotics in this river than in most other rivers [36], and the main factors that affected the antibiotic content were aquaculture, pharmaceutical wastewater, livestock discharges, domestic sewage, and sewage treatment plants [36,37]. Moreover, Wang et al. found a high level of ARGs pollution in the Weihe River, the largest tributary of the Yellow River [38]. Therefore, the inflow of pollutants upstream may lead to a high content of ARGs in Y1.

### 3.2. Distribution Pattern of ARG Subtypes in the Yellow River

Figure S1 indicates the distribution catachrestic of the ARG subtypes in the Yellow River. The average abundance of *sul*1 (10.94 RPKM) was overall maximal in the river. Furthermore, the gene content is in the top two highest in almost all samples (excerpts Y4 and Y6), which indicates the prevalence of the gene in the Yellow River. A similar condition is found on *sul*2, of which the average value is the second highest. *Sul*1 and *sul*2 have been reported to be ubiquitous in various media, as they can transfer horizontally with the help of mobile genetic elements (MGEs) [39,40]. Moreover, the wide bacteria hosts may contribute to the domination of these two genes [41,42].

The abundance of 3 Aminoglycoside genes (*ANT*(2′′)-Ia, *ANT*(3′′)-IIa, and *APH*(3′)-Ia) is the third, fourth and fifth highest among all the detected genes. These genes could locate on the plasmid or integron, which facilitates their transfer in the environment [43,44]. *EreA*2 is the most prevalent gene among MLS-ARGs, with a detection rate of 80% and an average abundance of 2.26 RPKM. *EreA*2 is a clinically relevant esterase and is therefore regarded as critical ARGs [45]. The conserved *ereA*2 sequences have been found in the integrons of various gammaproteobacteria, such as *Enterobacter aerogenes* and *Providencia stuartii* [46]. In conclusion, horizontal transfer contributes to the prevalence of the gene. In a low detection rate (23.3%) and content (average abundance is 0.41 RPKM), 3 carbapenems-resistant genes (*bla*_OXA-17_, *bla*_OXA-21_ and *bla*_GES-5_) are found. Carbapenems mediate broad-spectrum activity against pathogens and are regarded as one of the most important antibiotics in clinical care [47,48]. However, studies finding carbapenem-resistant genes and bacteria in the environment were reported in recent years [49]. Pathogens that carry carbapenem resistance genes are resistant to carbapenem, and the infections caused by these pathogens are difficult to treat. In conclusion, the emergence of carbapenem resistance genes in the Yellow River suggests that the use of carbapenem antibiotics should be further restricted.

There are 41 ARGs detected in Y1, and the number is obviously higher than that of Y10, of which the number of detected genes is the second-highest in all sample sites. The abundance of 35 genes is the highest in Y1. The above results indicate the ARGs pollution level in Y1 is the heaviest in the Yellow River of the Henan section, and the upstream input may be one of the important reasons.

### 3.3. Microbial Source Tracking Genes Content and Their Relationship with ARGs

**Figure 3:**
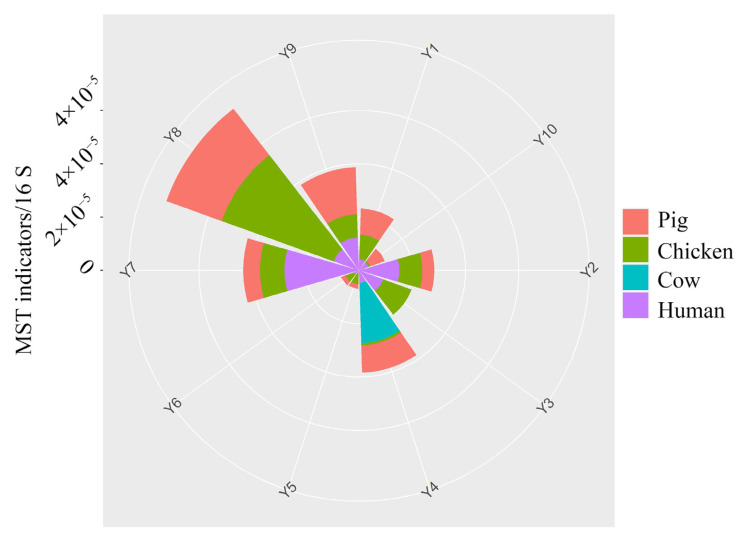
The relative abundance of MST indicators in the Yellow River. The chicken mt DNA content was divided by 10 to facilitate the visualization of MST indicators.

Three MST indicators (mtDNA of dog, sheep, horse) cannot be found in all samples, and the other indicators are shown in Figure 3. The chicken content indicator is the highest among all selected indicators, with a mean value is 1.01 × 10^−4^/16S copies. The Ministry of Agriculture of the People’s Republic of China reported that the number of poultries in Henan province in 2020 was 700 million. The large number of poultries leads to the high abundance of chicken indicators in the Yellow River. Human Mt DNA is the second-highest, with an average value is 9.16 × 10^−6^/16S copies. The high abundance of humans reflects that there exists an obvious anthropogenic impact in the Yellow River. Henan is a province with a large population. In 2021, the permanent population of the province was 98.83 million. Therefore, the Yellow River Basin is inevitably affected by human activities. The average content and detection rate of the pig is 7.97 × 10^−6^/16S copies and 90%, respectively. The number of pigs in Henan province was 38.87 million in 2020, which is the first in China. Therefore, the source marker gene of cattle is also common in the Yellow River Basin.

Spearman correlation analysis was performed to assess the relationship between MST indicators and ARG, and the results are shown in Table S1. There is no significant correlation between MSTs and ARGs. The result is quite different from the previous study, which indicated that fecal from humans and pigs might be one of the main sources of some ARGs and antibiotics [22]. The propagation of ARGs could be influenced by various parameters, including antibiotics, mobile genetic elements, and even various pollutants. Although the Henan province breeding industry is developed, the prohibition of antibiotics in the breeding industry in China has minimized the use of antibiotics in the breeding industry. Thus, the contribution of the breeding industry is reduced in the propagation of ARGs. A similar condition occurs in human beings. China has restricted the use of antibiotics in hospitals for a long time, which could minimize the contribution of human beings to the propagation of ARGs.

### 3.4. Characteristics of the Bacteria Communities

**Figure 4:**
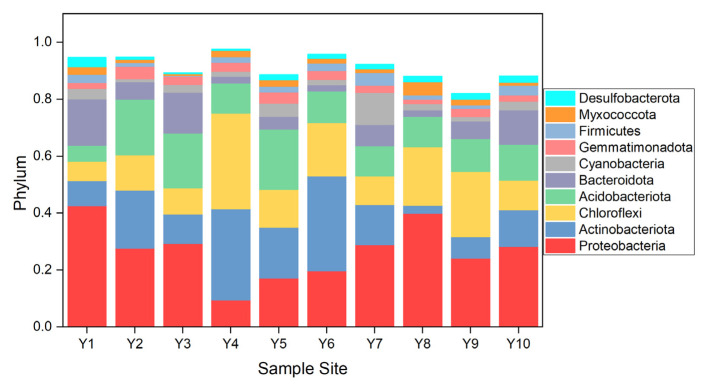
Top 10 bacteria at the phylum level in the Yellow River.

There are a total of 59 bacteria identified at the phylum level, and the top 10 phyla account for a large proportion (82.05–97.58%, Figure 4). *Proteobacteria*, *Actinobacteriota*, *Chloroflexi*, and *Acidobacteriota* are the dominant phyla in the Yellow River, which account for 26.72%, 15.99%, 15.79, and 13.26% of the total bacteria content on average, respectively. The aforementioned phyla are common bacteria that are ubiquitous in rivers, lakes, and marine bodies [50,51]. *Proteobacteria* are the most abundant and are mainly distributed in various environmental media that are contaminated by pollutants [52,53,54]. *Proteobacteria* encompass an enormous number of bacteria with a substantially high morphological, physiological, and metabolic diversity [55]. Consequently, bacteria from the phylum can survive under various environmental conditions.

The relative abundance of the top 100 bacteria is shown in Figure S2. In particular, numerous bacteria associated with denitrification, such as Thiobacillus, Flavobacterium, and *Arenimonas*, are the dominant bacteria in the Yellow River. These reduce nitrate and remove total nitrogen from the environment [56]. Therefore, the high content of TN and TP in sediment contributes to their prevalence. Some *Actinobacteriota* bacteria, including *RB41*, *Nocardioides*, and *Gaiella*, are prevalent in the Yellow River. These bacteria are generalist bacteria of *Actinobacteriota*, and have been found in a diverse number of habitats and conditions [57,58]. Another *Actinobacteriota* bacteria, named *Corynebacterium* (with the average value being 0.99%), which was initially defined as a pathogen in 1896 to accommodate mainly pathogenic species, could be found in almost all samples [59]. Among the known human pathogenic members of *Corynebacterium*, *C. diphtheria* is a notorious strictly human-adapted species and the causative agent of the acute, communicable disease diphtheria [59]. Similarly, *Pseudomonas* is one of the top 20 bacteria in the Yellow River, with an average value of 4.47%. *Pseudomonas aeurogenosa*, one of the members of *Pseudomonas*, is an opportunistic pathogen and is the most common bacterium associated with nosocomial infections and ventilator-associated pneumonia. The bacteria could cause high mortality in cystic fibrosis (CF) patients and immunocompromised individuals. Therefore, attention should be paid to the presence of *Corynebacterium* and *Pseudomonas* [60].

### 3.5. Relationship between ARGs and Bacterial Communities

**Figure 5:**
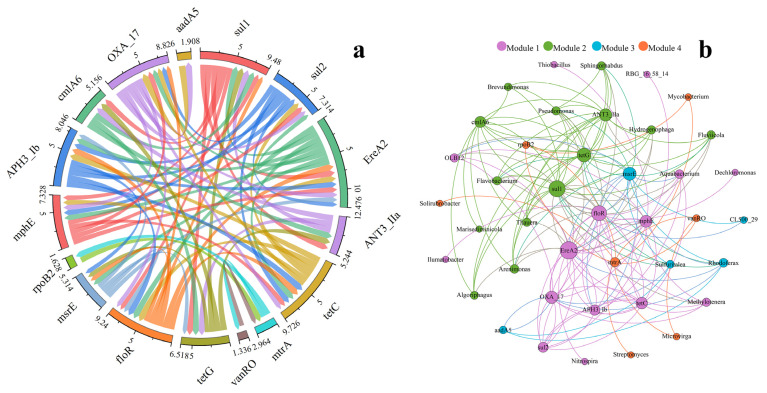
Relationship between ARGs and bacterial communities. (**a**) Spearman analysis results among ARGs. (**b**) Co-occurrence patterns among ARGs and bacteria (genus level).

In this study, Spearman’s correlation analysis was performed to explore the relationship among ARGs. The result is shown in Figure 5a. All the 16 ARGs involved in the correlation analysis are strongly correlated (r > 0.6, *p* < 0.05) with at least one ARG. Furthermore, there are 12 of the 16 ARGs strongly correlated with at least four other genes. The results demonstrate that there is a close relationship among the ARGs. Various ARGs could locate on the same mobile genetic elements, which could facilitate their diffusion. Moreover, the co-selection of various environmental factors could pose selective pressure to ARGs, which also contributes to their relationship.

In this study, network analysis was conducted to explore the co-occurrence pattern between ARGs and bacteria. Each connection represents a strong (r > 0.6) and significant (*p* < 0.05) relationship. The node size is proportional to the connection number; the larger the connection number, the larger the node size. The topology is divided into 4 modules, and ARG or bacteria in the same module had a closer correlation with each other compared to that of the other modules. The module size from large to small is Module 1 (14 nodes) = Module 2 (14 nodes) > Module 4 (6 nodes) = Module 3 (5 nodes). Numerous bacteria in Module 1 and Module 2 are prevalent bacteria in the Yellow River. For example, the abundance of Thiobacillus (Module 1) is the second highest in the Yellow River, and the abundance of Arenimonas (Module 2) is the eighth highest. Moreover, the prevalent ARGs, such as *sul*1 and *sul*2, are included in these two modules. Previous studies indicated that network analysis could be the way to explore the potential host of ARGs, and bacteria correlated to ARGs were the possible hosts of ARG [61,62]. The results of the network analysis of this study suggest that the dominated hosts contribute to the prevalence of ARGs. An opportunistic pathogen *Pseudomonas* can be found in Module 2, and *sul*1, *tet*G and *ANT*(3′′)-IIa are also included in the module. A previous study indicates that the ARGs in the same module could consider that these genes may locate in the same mobile genetic elements (MGEs) or be carried by the same bacteria. The result of this study implies that *Pseudomonas* in the Yellow River may be resistant to Sulfonamides, Tetracyclines, and Aminoglycosides, which may pose a risk to human health.

## 4. Conclusions

1. *Sul*1 and *sul*2 are the dominant ARGs in the Yellow River, which could be attributed to the huge usage of sulfonamides, horizontal gene transfer, and wide bacteria host of these two genes. Furthermore, the emergence of carbapenem-resistant genes indicates that the use of carbapenem antibiotics should be further restricted.
2. Animal and human feces are found in the Yellow River, while the prohibition of antibiotics in the breeding industry could minimize the contribution of the breeding industry to ARGs.
3. Numerous dominant bacteria are associated with denitrification, and the prevalence of some opportunistic pathogens (e.g., *Corynebacterium* and *Pseudomonas*) may pose an adverse health risk to humans.
4. There exists a close relationship among ARGs. Furthermore, *Pseudomonas* is the potential host of *sul*1, *tet*G, and *ANT*(3′′)-IIa, which may pose a risk to human health.

## 5. Perspective

This study determined the pollution level of antibiotic resistomes in the Henan section of the Yellow River and analyzed the contribution of fecal pollution to ARGs. Moreover, the network analysis was conducted in this study to explore the co-occurrence pattern between ARGs and bacteria. Antibiotic-resistant bacteria should be identified, and antibiotic susceptibility testing should be carried out in further studies to provide new insight into broad antibiotic resistance in the Yellow River.

## Data Availability

The data presented in this study are available on request from the corresponding author.

## Supplementary Materials

The following supporting information can be downloaded at: https://www.mdpi.com/article/10.3390/ijerph191610420/s1. Figure S1: The content of each detected ARG in sediments in the upper reaches of Huaihe river. Figure S2: The relative abundance of the top 100 bacteria in the Yellow River. Table S1: Primer information in the Yellow River. Table S2: The result of Spearman correlation analysis between MST indicators and ARGs.
