# Supplementary material for: The Sources and Potential Hosts Identification of Antibiotic Resistance Genes in the Yellow River, Revealed by Metagenomic Analysis
Source: Int J Environ Res Public Health. 2022 Aug 21;19(16):10420. doi: 10.3390/ijerph191610420 (PMC9408424; doi:10.3390/ijerph191610420)
Supplement: Supplementary file 1 [file ijerph-19-10420-s001.zip › ijerph-1842654-supplementary.pdf]

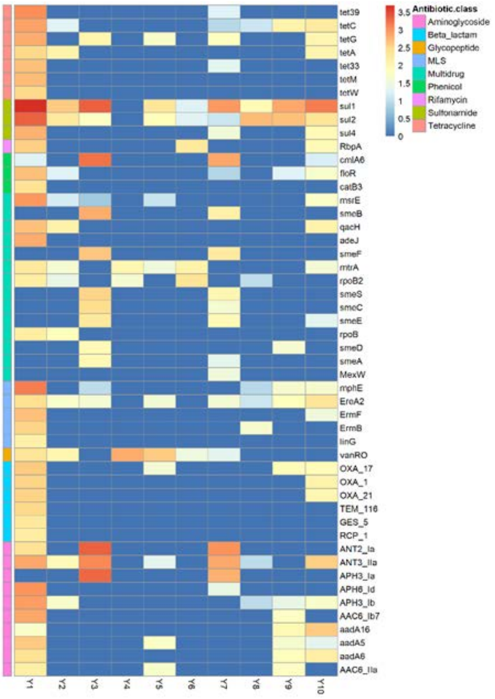

Figure S1. The content of each detected ARG in sediments in the upper reaches of Huaihe river

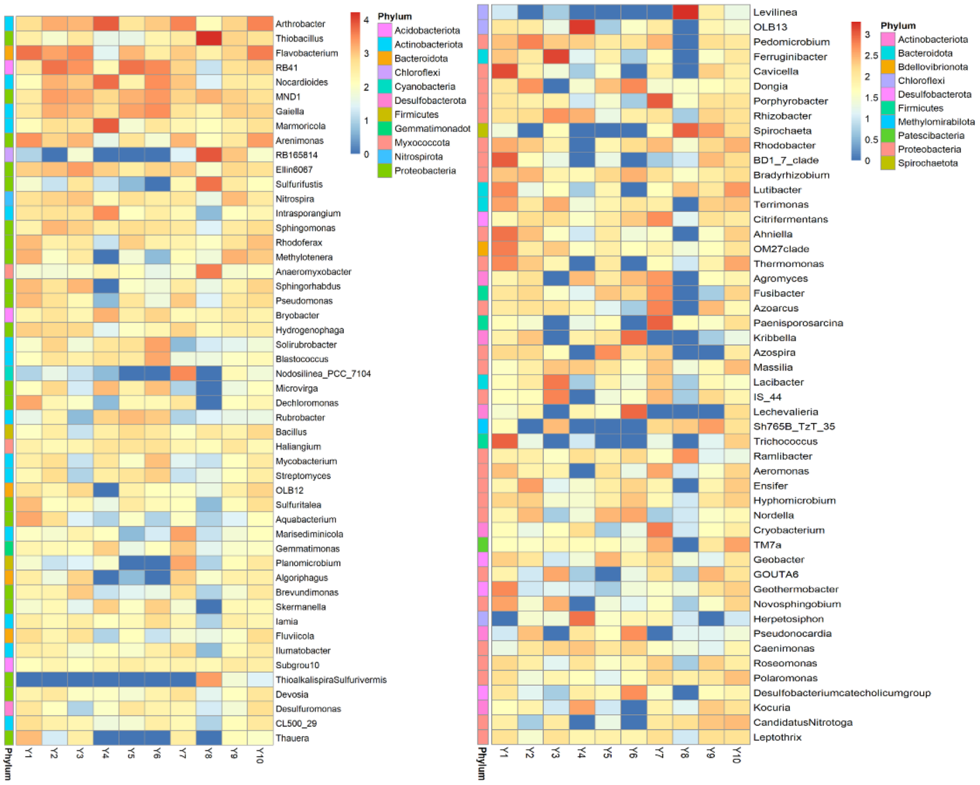

Figure S2. The relative abundance of the top 100 bacteria in the Yellow River

Table S1. Primer information in the Yellow River

Target gene	Primer sequence (5'-3')	Amplicon size (bp)	Reference
mtDNA-human-specific	F-AGT CCC ACC CTC ACA CGA TTC TTT<br>R-AGT AAG CCG AGG GCG TCT TTG ATT	185	Schill and Mathes, 2008
mtDNA – horse-specific	F-AGG AGC AAC AGT CAT CAC GAA CCT<br>R-AAA TGT ACG ACT ACC AGG GCT GTG	168	Schill and Mathes, 2008
mtDNA – cow-specific	F-AAT GCA TTC ATC GAC CTT CCA GCC<br>R-ACG TCT CGG CAG ATA TGG GTA ACA	173	Schill and Mathes, 2008
mtDNA – dog-specific	F-CCA CAG CAT TCA TGG GCT ATG TAC T<br>R-AGC TGC GAT GAT GAA AGG GAG GAT	200	Schill and Mathes, 2008
mtDNA –chicken-specific	F-TAG CCA TGC ACT ACA CAG CAG ACA<br>R-TTT GCG TGG AGA TTC CGG ATG AGT	103	Schill and Mathes, 2008
mtDNA –pig-specific	F-CGA CAA AGC AAC CCT CAC ACG ATT<br>R-TAG GGT TGT TGG ATC CGG TTT CGT	117	Schill and Mathes, 2008
mtDNA –sheep-specific	F-ACG CAT TCA TTG ATC TCC CAG CTC<br>R-TCG GCA AAT GTG GGT TAC AGA GGA	167	Schill and Mathes, 2008

Table S2. The result of Spearman correlation analysis between MST indicators and ARGs

mtDNA	*sul*1	*sul*2	*EreA*2	*ANT*3_IIa	*van*RO	*tet*C	*mtr*A	*mph*E
pig	-0.18788	0.381818	0.036474	-0.3988	-0.12506	0.468979	-0.1063	0.316789
chicken	0.333333	0.309091	0.085107	0.411074	-0.40645	0.056277	-0.57528	0.239208
human	0.151515	-0.00606	0.158055	0.141115	-0.33141	0.231363	-0.51275	-0.0194
mtDNA	*msr*E	*tet*G	*APH*3_Ib	*flo*R	*rpo*B2	*cml*A6	bla_{OXA17}	*aad*A5
pig	-0.35558	-0.35558	0.342649	0.200418	0.148697	-0.2936	0.198009	0.061451
chicken	-0.18749	0.187487	0.006465	-0.12284	-0.18749	0.375534	-0.17753	-0.16387
human	-0.31679	-0.20042	0.161627	0.200418	-0.22628	0.170697	-0.31408	-0.43698
